# Determination of Si/graphite anode composition for new generation Li-ion batteries: a case study

**DOI:** 10.55730/1300-0527.3507

**Published:** 2022-10-08

**Authors:** İlknur KALAFAT, Neslihan YUCA

**Affiliations:** 1 Enwair Energy Technologies Corporation, İstanbul, Turkey; 2 Faculty of Engineering and Natural Sciences, Electrical and Electronics Engineering, Maltepe University, İstanbul, Turkey

**Keywords:** Silicon-graphite anode, lithium-ion battery, specific capacity

## Abstract

Silicon with the properties of high capacity capability, moderate working potential, environmental sensitivity, and existence are the highly promising anode materials for lithium-ion batteries. Silicon anodes have disadvantageous properties and advantages like 300% volume change during lithium insertion and extraction process that can result in capacity fading and a shorter lifetime of the battery. In the literature, different optimizations of Silicon with different nanomaterials or composite materials, in different ratios, and with different binders and different procedures have been studied.

The physical mixing of silicon with carbon provides a good performance by combining the high lithium storage capacity of the silicon and the good mechanical and conductive properties of carbon. Binders are one of the other factors affecting the performance of the Si/C anodes. In this study, different ratios of silicon/graphite combinations were tested. The Si/C hybrid material provides an advantageous and efficient use for innovative lithium-ion anodes and available lithium-ion battery technology when the Si/C match performs a suitable combination of two material properties, such as the high lithium storage capacity of silicon and the conductive properties of carbon. This study is aimed to improve the performance of the cell by changing the amount of active material and polymer in the electrode by finding the most appropriate amount of active substance and binder polymer ratio in the electrode. The electrochemical result of the composition, which compensates for the problems caused by the volume expansion of the silicon by using less silicon, showed higher capacitive properties, as it exhibits better adhesion among these compositions with a higher binder ratio. This study resulted in more than 1000 mAh/g specific capacity after 100 cycles at C/3 rate and structural characterization of the samples before and after cycling provided information about the electrode content.

## 1. Introduction

Scientists have been working on developing new materials and designs for energy storage systems. Electrochemical storage is one of the most important technologies for electric vehicle applications. Moreover, the increase in portable electronic devices requires the development of energy storage devices with higher energy densities [1]. Lithium-ion batteries (LIBs) can meet this energy storage demand.

An LIB is mainly composed of an anode, cathode, separator, and a certain amount of electrolyte [2]. Cathode materials are generally lithium-containing metal oxides and anode materials include insert type materials (graphite, LTO (lithium titanate), etc.) and alloy-type materials. The separator which is used to prevent a short circuit between the cathode and the anode electrodes is mostly made of polyethylene (PE), polypropylene (PP), poly(tetrafluoroethylene) (PTFE), and poly (vinyl chloride) (PVC) [3]. The electrolyte that is based on a solution of dissolved inorganic lithium salts in a mixture of different organic solvents [4] should be a good ionic conductor and electronic insulator. Among such influencing materials, intensive research has been carried out to develop silicon anodes for enhancing the energy density of LIBs. The use of graphite combined with silicon particles is an effective orientation to improve the electrochemical performance of the Si-based anode. Graphite can be used not only to have a stable solid electrolyte interphase (SEI) but also to provide pores for silicon particles between graphite flakes. Silicon nanostructure dispersed in graphite matrix is designed to reduce the volume change of silicon electrode and decrease the reaction between silicon/electrolyte during charge/discharge. Compared to pure silicon, the use of silicon/graphite on the anode provides a better capacity gain [5].

In LIBs, graphite as a commercial anode material exhibits a good cycle performance, but in its use as an active substance alone, it offers a theoretical specific capacity of 372 mAh/g [6]. To obtain higher capacity LIBs, new generation anode materials with high capacity were investigated. The silicon has a high theoretical capacity of 3576 mAh/g which is 10 times higher than that of graphite and 20 times higher than LTO [7].

The anode prepared as a mixture of Si/graphite is expected to be homogeneous to provide low capacity loss. Furthermore, the aim is to decrease the instability of silicon due to the SEI formation by placing the silicon particles in the pores of the graphite matrix [8]. With the addition of graphite, there may be an improvement in the performance of the cell by decreasing the volume changes in the Si/graphite mixture. To obtain high capacity Si-graphite-based anodes, a higher capacity or conductivity can be created by adding another carbon source, as well [9]. Moreover, the addition of the organic polymer to the Si/graphite mixture serves to form a strong interconnection between graphite and Si and to provide a homogeneous distribution of Si particles on the graphite.

The various binders used in many silicon anode batteries are polyacrylic acid (PAA), poly(vinyl alcohol (PVA), alginate, carboxymethyl cellulose (CMC), and Polyvinylidene fluoride (PVDF). There is the volume expansion of the silicon material because of its weak van der Waals forces. Unfortunately, these binders cannot prevent problems of silicon anode. Methods to alter binder materials have been used to improve the performance of lithium-ion batteries, including those with silicon-based anodes. According to Table 1, researchers tried to improve silicon/graphite anode of electrochemical performance by modifying alginate and increasing the ratio of graphite. Kim et al. reported that 79.7% electrochemical efficiency was observed in the Si/Gr electrode prepared with alginate binder after 300 cycles at 0.5C [10]. Electrochemical performance of Si/Gr anode was improved by increasing the efficiency to 90% when using an alginate binder modified with re-DNA. In another research, the alginate used as a binder was modified with catechol [11]. The catechol interacts with silicon and improves surface properties. The Si/Gr electrode using alginate-catechol binder provided 55% better electrochemical performance than the silicon/graphite anode using only alginate binder as given in Table 1. Moreover, Gendensuren et al. obtained a flexible binder by exploiting the ionic interaction between Alginate and polyacrylamide (PAAm). At the same time, there is a covalent interaction between alginate and PAAm. This binder has been shown to significantly improve the cycling performance of the Si/C anode by improving the mechanical properties [12]. The alginate is a strong alternative for the binder with high mechanical properties. The PVA provides strong adhesion to the electrode surface [13]. It can be strong hydrogen bonds between the hydroxyl groups in PVA and active materials with another binder interaction. Combining PVA with alginate is an approach worth mentioning for Si-based anodes. Alginate can be modified using physical crosslinking with PVA, which makes them more effective in containing the volume expansion for more cycles and maintains better contact with all the anode materials, even if the silicon surface continues to crack. Both covalent crosslinking and supramolecular interactions in the binder maintain their intrinsic good binding properties and additionally enhance lithium-ion diffusion.

In this experiment, the alginate/PVA combination was used as a binder to provide good binding and flexibility [14]. Si/C mixture has better electrochemical properties by keeping high efficiency and good capacity. Here, we attempted to buffer the volume change of Si during the cycles. This study showed that graphite is successful in increasing the cycle life and performance of Si. In light of all these studies, Si/C anodes with low cost and relatively better performance can be used as suitable anodes for high-performance LIBs.

## 2. Materials and methods

**Figure 1:**
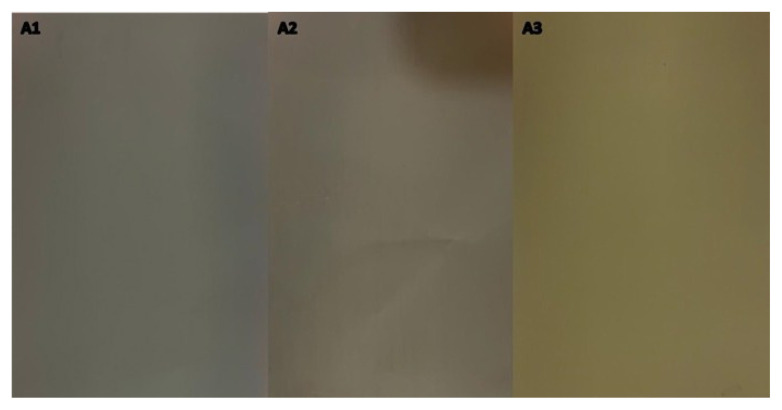
Images of as-prepared and dried sheets.

The anode was prepared with nanosilicon powder (particle size 30–50 nm, Nanostructured & Amorphous Materials, Inc.) and graphite (particle size 1–5 micron, Tob New Energy Inc.). The electrolyte was purchased from BASF (company), including 1.2 M LiPF_6_ (lithium hexafluorophosphate) salt in ethylene carbonate, diethyl carbonate, and fluoroethylene carbonate (EC: DEC: FEC = 1.16: 1.16: 1 w/w). Sodium alginate/PVA (9: 4) polymers were used as binders which were purchased from Sigma-Aldrich. The polymers were dissolved in water and the carbon additives and anode active materials were added, respectively. The silicon and graphite were mixed during slurry preparation. Three different ratios were tried as active material and binder and different silicon graphite ratios and the ratios are shown in detail in Table 2. The mixture was stirred for 1 h in an ultrasonic homogenizer and for 2 h in a magnetic stirrer, and the electrode slurry was coated in copper foil using a doctor blade coating method. The prepared electrode was dried in a vacuum oven at 100 °C for 15 h. Prepared and dried electrodes are shown in Figure 1. Electrodes were characterized by SEM, EDX, and XRD measurements. X-ray diffraction (XRD) patterns were obtained using Bruker D8 Advance X-Ray Diffractometer (Cu Kα radiation (λ=0.154 nm). The morphologies of electrodes were examined by Zeiss Ultra Plus Field Emission Scanning Electron Microscope (FE-SEM). Elemental analysis was carried out by energy-dispersive X-ray spectroscopy (EDX) detector connected with SEM. Coin cells were prepared against Li metal as reference electrodes. Cells were tested at 1.2–0.01 V at C/25 in 2 cycles and C/3 for the following cycles. Cyclic voltammetry (CV) experiments and impedance (EIS) measurements were performed using Gamry Interface 3000. Cyclic voltammetry (CV) was performed in the potential range between 0.01 and 1.2 V at a scan rate of 0.1 mV/s and electrochemical impedance spectroscopy (EIS) was performed between 100 kHz and 10 MHz.

## 3. Results

**Figure 2:**
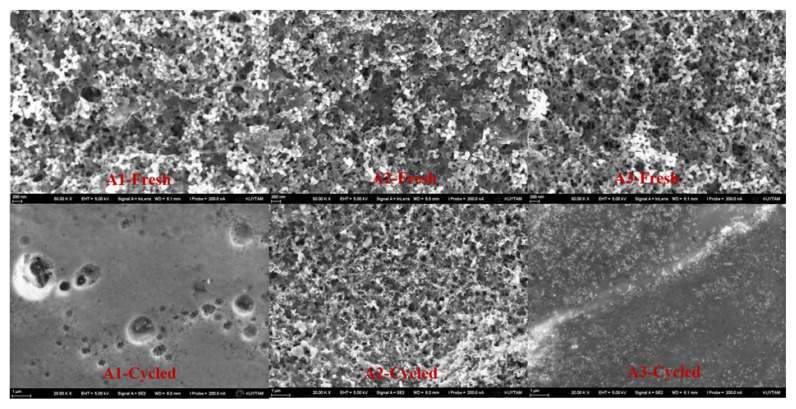
SEM images of A1, A2, and A3 samples before and after cycling.

Graphite and silicon, which are used as an anode active material in lithium-ion batteries, have various advantages and disadvantages. Nanosized silicon which has 10 times higher specific capacity than graphite shows the volumetric expansion problem at the level of 400% while it is 10% in graphite. This volumetric change results in cracks on the electrode structure and also the formation of an unstable SEI layer. This unstable SEI layer of silicon anode causes lithium depletion and performance degradation. Figure 2 shows expansion and shrinkage due to lithium insertion-extraction during charge-discharge, schematically. To benefit from the stable performance of graphite and the high specific capacity of silicon, silicon/graphite anodes in the ratios given in Table 2 were prepared and their electrochemical performance was investigated.

Figure 2 shows SEM results of A1, A2, and A3 samples before and after cycling. It was observed for each sample that electrodes have well-distributed silicon active material yielding a network provided by polymer as binder and CNT as carbon additive. The graphite particles could not be identified at 200K× magnification since 30–50-nm-sized silicon particles coated on the surface. However, in the SEM images at the lower magnification given in Supplementary data (S1), they are seen clearly. After the electrodes were cycled, SEM measurements were performed again to look through the electrode structure. Even at lower magnifications, it was not available to get a clear image for samples A1 and A3 which can be explained by a thick SEI layer on the electrode surface while the silicon particles can be still distinguishable for sample A2 after cycling. More SEI layers can be the result of more silicon content in samples A1 and A3. To identify the silicon/carbon phases and content XRD and EDX measurements were performed for the fresh and the cycled electrodes.

**Figure 3:**
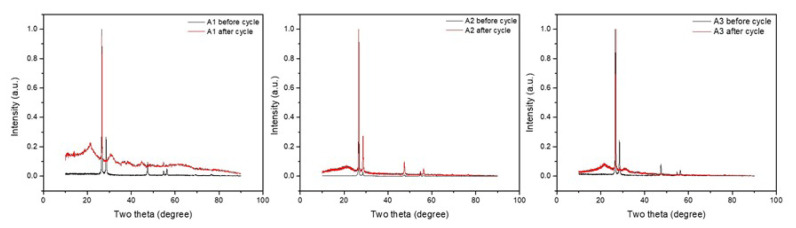
XRD results of A1, A2, and A3 samples before and after cycling.

Figure 3 shows the XRD patterns of the samples containing silicon and graphite before and after cycling. Pattern numbers and detailed two theta degrees (2θ°) are given in Supplementary data as S2. According to XRD results, the A1 sample before the cycling shows the diffraction pattern of cubic Si (JCPDS card no. 01-075-0589) which is the peaks shown as 28°, 47°, 56°, 69°, 76°, and 88°. It shows itself at 28° next to the most intense peak. Other matching peaks are less intense. The peak we see at 25° as the most intense peak belongs to graphite [16]. The peaks around 25° and 54° are the peaks of graphite representing two hexagonal phases. The fact that the graphite in the composite has more intense peaks may be due to the relatively higher graphite content. There was no chemical reaction between silicon and carbon. No peaks corresponding to SiC were detected. When the XRD patterns are examined for the A2 sample before the cycle, the diffraction pattern of cubic Si (JCPDS card no. 01-077-2110) is the peaks shown as 28°, 47°, 56°, 69°, and 76°. Of these, the most intense peak appears at 28°, like the Si diffraction in the A1 sample. Other matching peaks are less intense. Likewise, with A1, the peak we see at 25° as the most intense peak belongs to the rhombohedral carbon. This carbon roughly corresponds to graphite. For the A3 sample before the cycle, the XRD patterns have similar diffractions to the silicon and graphite diffraction in the A1 sample. When the after cycle XRD patterns are examined; the 25° graphite peak is obvious, the Si diffraction was indistinct and replaced by silicon oxide. Silicon oxide diffractions are initially observed between 20° and 25° bands. There are different silicon oxide patterns for each after cycle sample. From this point of view, it can be said that different phases occur during the conversion for each sample. Comparing the after cycle peaks, it appears that the nonintense Si diffraction of the A2 sample is obvious, while the other Si diffractions are close to disappearing. From this, it can be predicted that the silicon in the A2 sample undergoes less deformation due to the binder effect. The significant graphite peaks indicate that the silicon is not completely covered on the surface of the graphite. The peaks corresponding to silicon oxide appear after each pattern cycle, indicating that silicon is oxidized under current. All peaks of the after cycle visible at 28° are attributed to the width of silicon oxide. With a theoretical capacity of 372 mAh g^−1^, the reversible capacity of graphite after 50 cycles with commercial binder PVDF is about 350 mAh g^−1^, and the capacity retention rate is 95%. The Si/graphite composite, on the other hand, is about 390 mAh g^−1^ with lower capacitance retention of 85%. The results show that the cyclic stability of graphite decreases after silicon addition, but its capacitive value is much better than that of pure graphite anode. Silicon/carbon anode prepared with PVA-alginate binder yielded nearly 3 times better results when it is compared to conventional graphite electrodes. Thick to thin (400–50 μm) coatings were made to control adhesion with the addition of PVA. It showed good adhesion on all coatings. The X-ray diffraction patterns in Figure 3 clearly show a sustained increase in intensity for the graphite peaks at the postcycle electrodes. XRD patterns of A1, A2, and A3 samples including silicon and graphite given in Supplementary data (S2), they are seen clearly. The losses of the other peaks indicate that the reaction has occurred at the interface with the electrolyte. Here we show that the peak density gain for Li-C-Si is mainly due to the chemical reaction between the lithium silicon-graphite anode and the electrolyte, which is activated after decomposition of the SEI layer. This finding ascribes to the SEI thickness in the SEM findings, with the effect of the stability of the SEI layer on the electrochemical performance of lithium-ion batteries using graphitic anodes.

**Figure 4:**
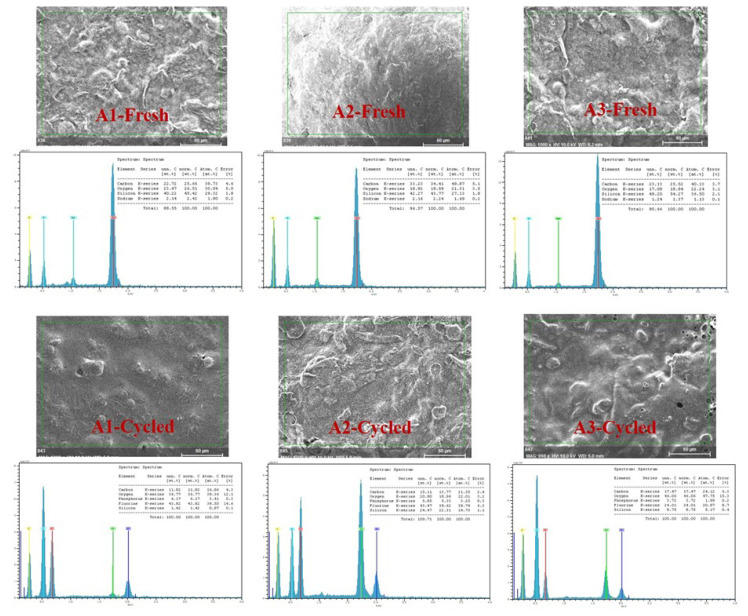
EDX results of A1, A2 and A3 samples before and after cycling.

In Figure 4, EDX measurement provided a clear understanding of the composition of each sample for before and after cycling processes. Before cycling, C, O, Na, and Si peaks were observed. Na peak comes from the alginate binder which is in sodium alginate salt form. After cycling, all the samples showed C, O, F, Si, and P peaks. F and P peaks can be explained as the component of the SEI layer.

**Figure 5:**
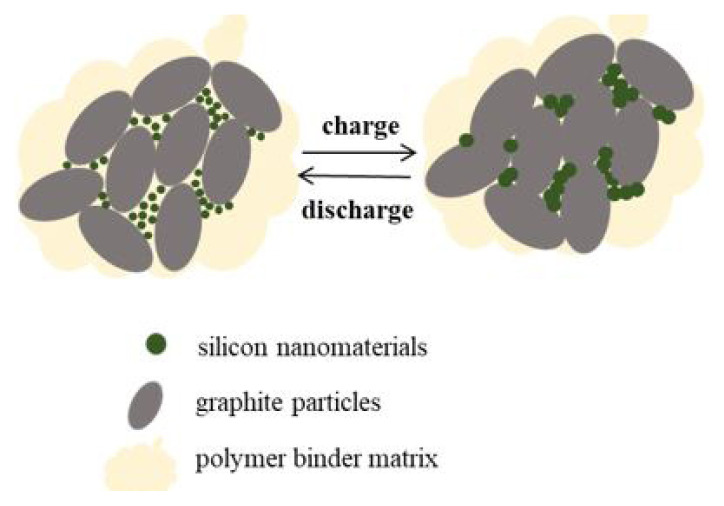
Schematic representation of the volumetric change in the active material during charge-discharge.

When the structure of the Si/graphite electrodes as shown in Figure 5 is examined, it is seen that the spherical silicon nanoparticles are placed between the micron-sized graphite flakes. Porosity between particles varies depending on the shape, pattern, and size of the materials [15]. The graphite particles are micron-sized and are thought to have sufficient space in the volumetric expansion by locating silicon nanoparticles in the spaces between these particles. Thus, both the stability of the graphite and silicon without creating cracks after volumetric expansion allowed the capacities to be reached higher than the theoretical capacity of graphite in all samples as seen in Table 3.

**Figure 6:**
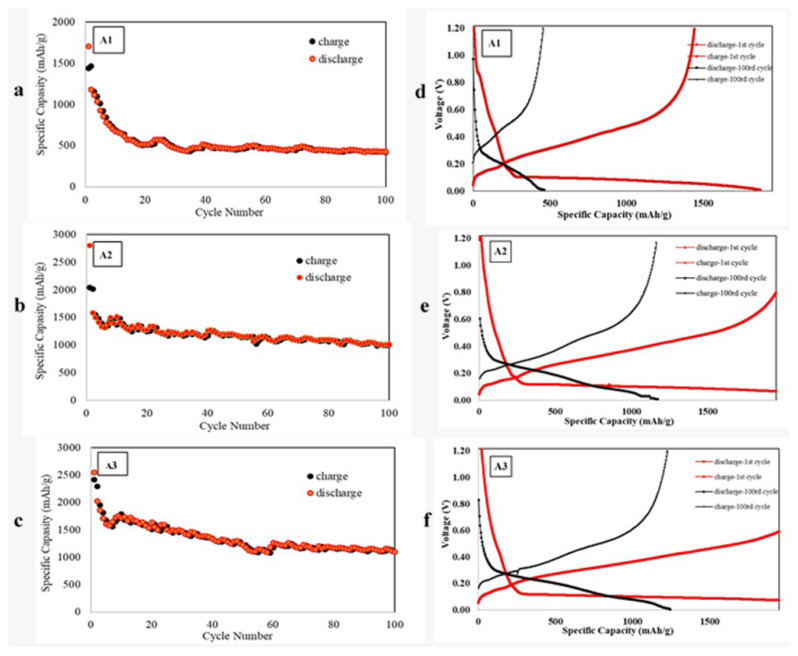
a-c) Capacity-cycle graphs of A1, A2, and A3 samples, respectively, d-f) voltage-capacity graphs of A1, A2, and A3 samples, respectively.

In the preparation of silicon/graphite anode, experiments were made using equal amounts of active material and the active material was tested to be 60% and 70% of the total electrode. Figure 6a shows the cycle-capacity graph of the A1 sample, which contains the same percentage of silicon/graphite active material (50:50) and 70% of the electrode is the active material. Silicon: graphite: binder: carbon additive was used at the ratio of 35%: 35%: 20%: 10%. The sample with an initial capacity of 1705 mAh/g was decreased to 1180 mAh/g in the second cycle. The sample has capacity decay at the first 20 cycles, and at the end of 100 cycles, it has a capacity of 427 mAh/g. In Figure 6b, when the A2 sample, where the 60% of the electrode is active material, was examined, silicon: graphite: binder: carbon additive was used at the ratio of 30%: 30%: 30%: 10%. It was observed that the initial capacity was 2801 mAh/g and it was decreased to 1587 mAh/g in the second cycle. Capacity drops in the A2 sample were slower than in the A1 sample and 1013 mAh/g capacity was obtained at the end of 100 cycles.

The major difference in the electrochemical behaviors of A1, A, and A3 was analyzed by the charge-discharge cycling measurements. The typical charge-discharge curves of samples are shown in Figures 6a–6c. In the voltage range of 0.01–1.2 V at C/3, samples delivered an initial discharge specific capacity of 1705, 2801, and 2546, respectively, whereas the samples exhibited the capacity at 100th cycle as 427, 1013, and 1094, respectively. Thus, A3 with the higher silicon content exhibited a much better initial performance than A1 and A2.

In this study, it is planned to use another ratio that aims to decrease the amount of active material in the electrode and increase the binding polymer. Here we aim to have increased the capacity by increasing the amount of silicon in the electrode. The electrochemical results of the A3 sample which has a silicon/graphite ratio of 70/30 and the active material content in the electrode as 60% are given in Figure 6c. Silicon: graphite: binder: carbon additive was used at the ratio of 42%: 18%: 30%: 10%. Thus, the binding ratio for A2 and A3 was kept constant and the effect of the active material could be compared on their electrochemical performance. The voltage-capacity graphs of samples in Figures 6d–6f show results for the first and 100th cycle. The A3 sample with a first cycle capacity of 2546 mAh/g provided a specific capacity of 1104 mAh/g in the second cycle and 1094 mAh/g in the 100th cycle. When A2 and A3 samples were compared, similar capacities were observed after 100 cycles. However, the higher silicon active material ratio increases the volumetric capacity by providing both lightweight and thinner electrodes. Table 3 shows the results and electrode properties.

**Figure 7:**
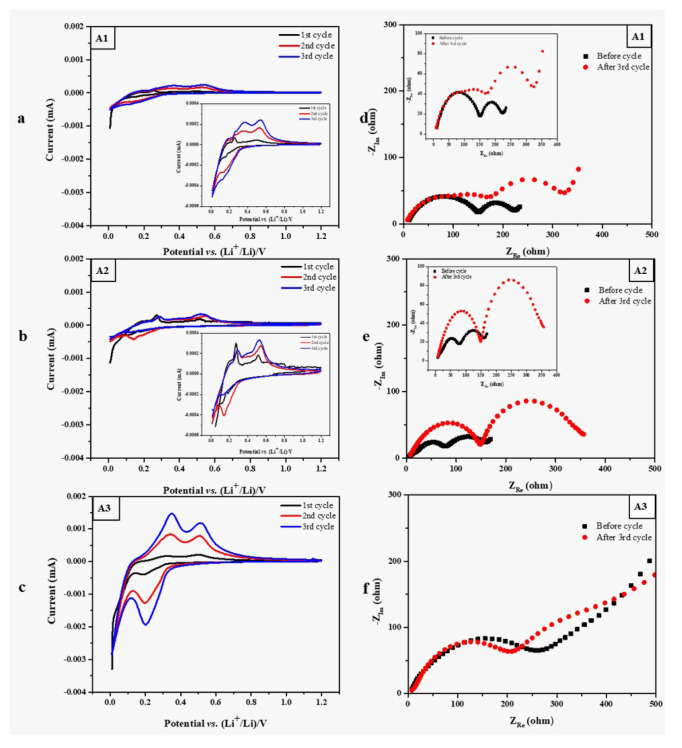
a-c) CV graphs of A1, A2 and A3 sample, respectively d-f) EIS graphs of A1, A2 and A3 sample, respectively. CV graphs show the first 3 cycles and EIS graphs belong to before and after cycling of samples.

Figure 7 shows the cyclic voltammetry and electrochemical impedance spectroscopy measurement results of the samples. In Figure 7a, the first cycle of the CV profile of A1, the peak at 0.19 V corresponds to the conversion of a-Si to the Li_x_Si phase in the cathodic branch that is similar to the A3 sample. The two peaks at around 0.3 and 0.5 V for all samples in the anodic branch correspond to the delithiation of a-Li_x_Si to a-Si. The red and blue lines in the figures show the second and third cycle CV profile of electrodes; that redox peak has a small shift for A1 and A2 while it is almost present at similar positions for A3. CV graphs show that the current begins to increase below 1.0 V, corresponding to the SEI formation and lithium reacts with silicon, but the current is much reduced during the second cycle. The lithium de-alloying process begins at about 0.5V. The cyclic voltammetry plots agree with the charge-discharged curves shown in Figures 7a–7c. It can be seen from the cyclic voltammetry results in Figures 7a–7c that the second peak of the A1 and A2 oxidation curve supports the formation of silicon oxide. It is possible to see the lithium-ion transfer and interphases in the impedance measurements shown in Figure 7. While the resistance of A1-fresh is 149.6 ohm, the resistance of A1-cycled is 150.8 ohm. While the resistance of A2-fresh is 112.2 ohm, the resistance of A2-cycled is 163.6 ohm. While the resistance of A3-fresh is 98.7 ohm, the resistance of A3-cycled is 118.8 ohm. Violent phase or magnitude changes can be observed with the Bode plot.

Figures 7d–7f show the typical impedance spectra for cells, as well. In combination with the cyclic voltammetry measurement, the impedance was measured before cycling and after the potential scans to the desired states, such as at 1.2 V (desired state), following the initial 3rd cycle for the cells. The semicircles can be assigned to the charge transfer impedance of the electrodes. In the high-frequency and medium-frequency regions, the semicircles are assigned to the SEI and impedance of the interfacial charge transfer, respectively. The low-frequency region is referred to as the impedance of Li-ion diffusion. After 3 cycles, A1 and A2 samples had two semicircles while the A3 sample has one semicircle. This can be explained by more stable SEI layer with high Silicon content in which the electrode is fabricated with a suitable binder that increases the electrode structure integrity providing a higher electron transfer. This is also consistent with the cycling performance of A3 which has a higher specific capacity than A1 and A2.

**Figure 8:**
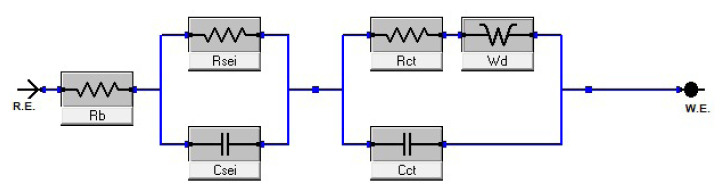
Equivalent circuit model diagram of impedance spectroscopy.

The impedances of cells were measured before it was put into cycling testing and after 3rd cycle. The equivalent circuit used in impedance measurements is given in Figure 8. In this model, R_ct_ is the charge transfer resistor, R_sei_ is the anode electrolyte interface resistance and R_b_ is the internal resistance of the rest of the equipment. It can be said that the closer the Warburg constant in the circuit model is to 45°, the better the ion diffusion. The Nyquist plot offers the convenience of analyzing possible mechanisms or governing events in an equivalent circuit model system. EIS patterns’ parameters of A1, A2, and A3 samples including silicon and graphite given in Supplementary data (S3), patterns’ values, errors, and goodness of fit are seen clearly.

**Figure 9:**
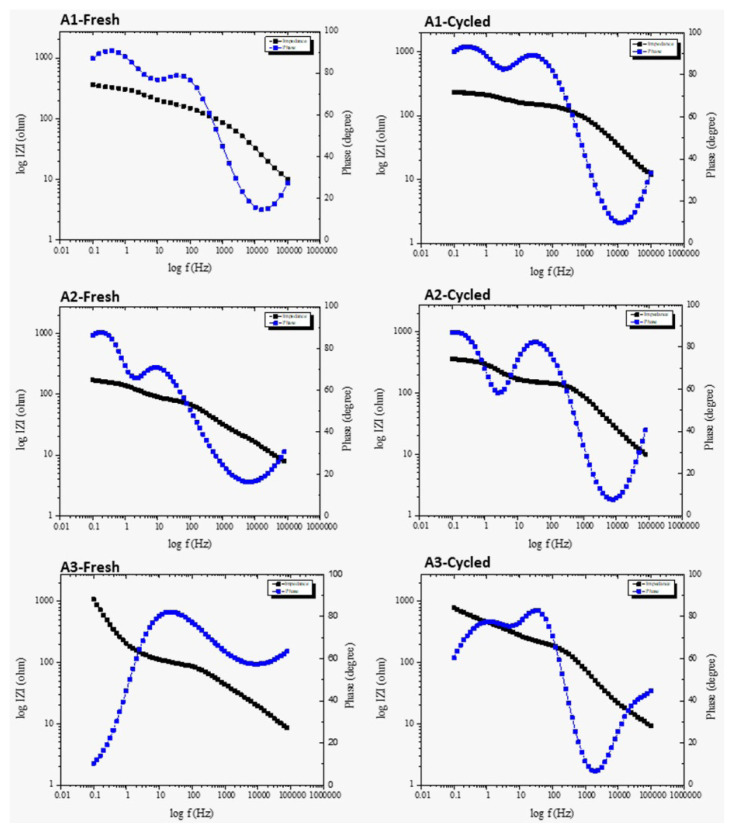
Bode diagrams of A1, A2, and A3 samples before and after cycling.

Figure 9 shows that there is not much phase change between fresh and cycled. If the bode diagram of each sample after the cycle is examined, the frequency and impedance decrease strongly with relaxation in the mid-low frequency region. This relaxation is attributed to the silicon oxide layer. With the decrease at the end of the frequency range, it can be said that Li^+^ ions or other species are formed on the electrode surface.

## 4. Discussion

The pure Si anode is not remarkable in industrial applications due to its short cycle life and rapid loss of capacity. However, there are ways to take advantage of the high capacity it offers. With the addition of a certain amount of commercial graphite, it is possible to stabilize the lifetime of cell and achieve much higher capacity than pure graphite. However, the binder to be selected must be successful against silicon and cell problems. The binder is responsible for the contact integrity between the active material, the conductive additive and the current collector. The PVA-alginate binder, which has functional groups that prevent the SEI layer from becoming unstable and form the homogeneous layer at the interface between the active material and the electrolyte, will reduce the silicon electrode surface crack problem by using strong supramolecular interactions with Si particles. In order to provide long cycle requirements, the binder content should be reduced and the amount of active ingredients should be increased. Addition of graphite to balance silicon is promising in terms of capacitive properties, as can be seen from the test results. A novel polymer binder synthesized via in situ cross-linking of water-soluble polyvinyl alcohol (PVA) and alginate precursor, is applied as a functional network binder to enhance the electrochemical performance of silicon/carbon anode. The Si/C anode with PVA-Alginate binder exhibits high specific capacity in the initial cycle. This electrochemical property is ascribed to the reversibly-deformable polymer network and the binder’s strong adhesion to the silicon particles. This low-cost and eco-friendly polymer binder has great potential to be used for silicon anodes in next-generation Li-ion batteries.

With a theoretical capacity of 372 mAh g^−1^, the reversible capacity of graphite after 50 cycles with commercial binder PVDF is about 350 mAh g^−1^, and the capacity retention rate is 95%. The Si/graphite composite, on the other hand, is about 390 mAh g^−1^ with a lower capacitance retention of 85% [17]. The results show that the cyclic stability of graphite decreases after silicon addition, but its capacitive value is much better than that of pure graphite anode. Silicon/carbon anode prepared with PVA-alginate binder yielded nearly 3 times better results when it is compared to conventional graphite electrodes.

The effect of the Si/C ratio in an anode composition for LIBs was investigated in this study. The results showed that increasing polymer content provided a more flexible structure for materials and the electrochemical performance exhibited better results. On the other side, higher Si content with higher binder content also showed increasing capacity by providing thinner electrodes which is important for portable electronics and electric cars in terms of volumetric capacity.

## Supplemantary Data

S1 SEM images of A1, A2, and A3 samples at lower magnification.

**S2:** XRD patterns of A1, A2, and A3 samples including silicon and graphite.

Sample name, (JCPDS no.) and two theta degrees	A1	A2	A3
**Two theta (before cycling)**	**Silicon** (01-075-0589) 28.441, 47.304, 56.125, 69.136, 76.377, 88.036	**Silicon** (01-077-2110) 28.508, 47.416, 56.259, 69.312, 76.583	**Silicon** (00-027-1402) 28.441, 47.304, 56.125, 69.136, 76.377, 88.036
	**Graphite** (01-075-2078) 26.611, 54.810	**Carbon** (00-003-0401) 26.190, 54.580	**Graphite** (01-075-2078) 26.611, 54.810
**Two theta (after cycling)**	-	**Silicon** (01-077-2110) 28.508, 47.416, 56.259, 69.312, 76.583	**Silicon** (01-080-0018) 28.652
	**Graphite** (01-075-2078) 26.611	**Carbon** (00-026-1080) 26.605, 47.304, 54.792	**Graphite** (01-075-2078) 26.611, 54.810
	**Silicon Oxide** (01-078-1254) 26.804	**Silicon Oxide** (01-083-2470) 22.02, 56.046, 69.168	**Silicon Oxide** (00-029-0085) 21.981

**S3:** EIS patterns’ parameters, values, and errors of A1, A2, and A3 samples including silicon and graphite.

Samples, (cycle state) and goodness of fit	Parameters	Value	Error	Unit
**A1 (before cycle)** **goodness of fit** **32.49e-6**	R _b_	6.168	372.4e-3	ohm
	Wd	38.78e-3	9.123e-3	S*s ^(1/2)^
	R _sei_	149.6	2.863	ohm
	R _ct_	55.78	7.213	ohm
**A1 (after cycle)** **goodness of fit** **863.6e-18**	R _b_	3.523	493.0e-3	ohm
	Wd	14.83e-3	3.015e-3	S*s ^(1/2)^
	R _sei_	150.8	10.60	ohm
	R _ct_	153.4	24.00	ohm
**A2 (before cycle)** **Goodness of fit** **1.590e-3**	R _b_	2.898	309.5e-3	ohm
	Wd	41.41e-3	8.591e-3	S*s ^(1/2)^
	R _sei_	108.9	5.137	ohm
	R _ct_	38.72	7.676	ohm
**A2** ( **after cycle)** **goodness of fit** **797.8e-6**	R _b_	6.069	231.3e-3	ohm
	Wd	28.17e-3	7.583e-3	S*s ^(1/2)^
	R _sei_	163.6	9.892	ohm
	R _ct_	152.9	2.218	ohm
**A3** ( **before cycle)** **goodness of fit** **37.68e-3**	R _b_	5.144	398.4e-3	ohm
	Wd	2.212e-3	27.71e-6	S*s ^(1/2)^
	R _sei_	23.04	2.863	ohm
	R _ct_	36.60	3.932	ohm
**A3 (after cycle)** **goodness of fit** **2.565e-3**	R _b_	6.182	6.182	ohm
	Wd	2.604e-3	110.2e-6	S*s ^(1/2)^
	R _sei_	230.8	6.426	ohm
	R _ct_	111.6	16.41	ohm

## Figures and Tables

**Table 1:** A comparison table including the performance of different ratios of Si/C anode containing alginate and/or PVA as binders.

Binder name	Active material: binder: carbon additive (%)	Silicon: carbon (%)	Electrochemical results (efficiency, capacity)	Reference
Alginate	60:20:20	50:50	79.7%, 510.6 mAh g^−1^ after 300 cycles at 0.5C	Kim et al. [10]
Alginate	60:20:20	10:90	79.7%, 1.73 mAh cm^−2^ (Si/Gr loading to 6.2 mg cm^−2^) after 120 cycles at 0.6C	Kim et al. [10]
reDNA-alginate	60:20:20	50:50	90.0%, 593.7 mA h g^− 1^ after 300 cycles at 0.5C	Kim et al. [10]
reDNA-alginate	60:20:20	10:90	93.5%, 2.29 mAh cm^−2^ (Si/Gr loading to 6.2 mg cm^−2^) after 120 cycles at 0.6C	Kim et al. [10]
Alginate	60:20:20	60:40	62.8%, 522 mA g^− 1^ after 150 cycles at 1C	Ryou et al. [11]
Alginate-catechol	60:20:20	60:40	84.5%, 805.8 mA g^− 1^ after 150 cycles at 1C	Ryou et al. [11]
Alginate–PAAm	74:15:9	75:25	72.8%, 836 mA h g^− 1^ after 100 cycles at 1C	Gendensuren et al. [13]
PVA	85:10:5	30:70	620 mA h g^− 1^ after 50 cycles at 0.5C	Oh et al. [14]
PVA-alginate	70:20:10	50:50	427 mA h g^− 1^ after 100 cycles at 0.1C	This study
PVA-alginate	60:30:10	50:50	1013 mA h g^− 1^ after 100 cycles at 0.1C	This study
PVA-alginate	60:30:10	70:30	1094 mA h g^− 1^ after 100 cycles at 0.1C	This study

**Table 2:** Electrode and active material ratios.

	Active material: polymer binder: (CB:CNT)	Silicon: graphite
**A1**	70:20:(7.5:2.5)	50:50
**A2**	60:30:(7.5:2.5)	50:50
**A3**	60:30:(7.5:2.5)	70:30

**Table 3:** Capacity results and properties of electrodes.

Electrode name	1st cycle (mAh/g)	100th cycle (mAh/g)	100th cycle (mAh/cm^2^)	100th cycle (mAh/cm^3^)	Electrode weight (mg)	Electrode thickness*(μm)
**A1**	1705	427	0.415	188.63	0.875 Si+ 0.875 C	22
**A2**	2801	1013	1.9	1000	0.522 Si+ 0.522 C	19
**A3**	2546	1094	0.87	511.76	1 Si+ 0.432 C	17

* Electrode thickness without copper foil.
