# Supplementary figures and images for: Determination of Si/graphite anode composition for new generation Li-ion batteries: a case study
Source: Turk J Chem. 2022 Oct 8;46(6):2112–22. doi: 10.55730/1300-0527.3507 (PMC10446933; doi:10.55730/1300-0527.3507)

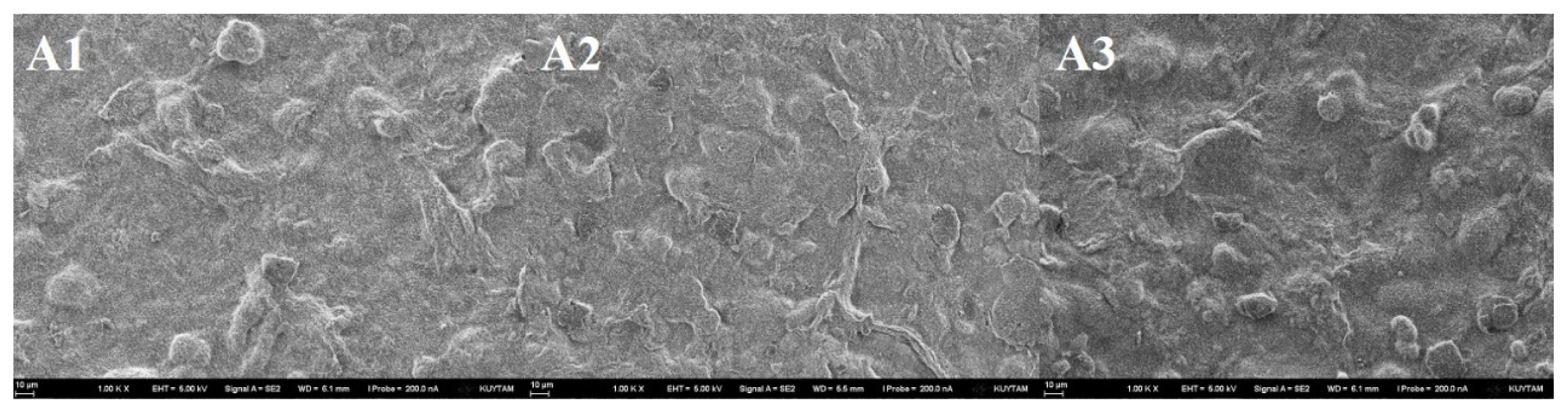

Supplement: S1 — SEM images of A1, A2, and A3 samples at lower magnification. [file turkjchem-46-6-2112s1.tif]
